# P06 Pharmacokinetics and renal accumulation of VRP-034

**DOI:** 10.1093/jacamr/dlac004.005

**Published:** 2022-02-16

**Authors:** Kamlesh Kumar Vishwakarma, Rahul Chilbule, Manu Chaudhary, Arun Sharma, Saransh Chaudhary, Anmol Aggarwal

**Affiliations:** Venus Medicine Research Centre, India; Venus Medicine Research Centre, India; Venus Medicine Research Centre, India; Venus Medicine Research Centre, India; Venus Medicine Research Centre, India; Venus Medicine Research Centre, India

## Abstract

**Background:**

Nephrotoxicity is a dose-limiting factor for polymyxin B (PMB), a last-line therapy for MDR Gram-negative bacterial infections. The majority of the filtered PMB undergoes extensive tubular reabsorption leading to significant accumulation of the drug in tubular cells, causing renal tubular damage. We have previously reported that VRP-034, a novel PMB formulation, attenuates this damage (Roy *et al.*^1^). The objective of this study was to characterize the pharmacokinetics (PK) and renal accumulation of VRP-034 versus marketed PMB.

**Materials and methods:**

A total of 19 Sprague-Dawley rats were used in the study. For PK evaluation, 10 rats were administered a single subcutaneous dose (6 mg/kg) of either VRP-034 or marketed PMB. Serial plasma samples were collected up to 24 h and assayed for major PMB components (PMB B1, B1-I, B2) using a validated LC-tandem MS method. PK parameters were calculated using noncompartmental analysis. For evaluating renal deposition, nine rats (*n = *3 each group) were administered VRP-034 or marketed PMB at a dose of 6 mg/kg/8 h for 48 h or normal saline (control). After 48 h, rats were euthanized and kidneys were excised by making a midline incision, washed with saline, homogenized in 1:9 w/v 0.1 M phosphate buffer (pH 7.4) and the homogenate was used for determining PMB concentration. All values are expressed as mean ± SEM.

**Results:**

The plasma pharmacokinetic parameters for both VRP-034 and marketed PMB were found similar [AUC_0–24_: 54.3 ± 4.7 μg/mL·h versus 51.5 ± 1.6 μg/mL·h; *C*_max_: 4.73 ± 0.6 μg/mL versus 5.02 ± 0.5 μg/mL; *T*_½_: 5.95 ± 0.5 h versus 5.6 ± 0.3 h]. In the renal deposition study, a 40% reduction (*P<*0.05) in renal deposition of PMB was found in VRP-034 treated rat kidneys compared with marketed PMB group after 48 h of treatment.

**Conclusions:**

The results highlight that while the plasma PK parameters remained unchanged, a marked reduction in PMB renal deposition was observed in VRP-034 group and likely explains the previous reports of attenuated PMB-induced kidney injury with VRP-034.
